# Acute Inflammatory Pericarditis following First Dose of COVID-19 Vaccine (AstraZeneca)

**DOI:** 10.1155/2022/9509023

**Published:** 2022-11-18

**Authors:** Mohammed Alshaikh, Abdullah Muharib, Radhi Alshehri, Abdulaziz Alshoaibi, Mohammed Qattea, Shahad AlOtaiby

**Affiliations:** ^1^Medical Imaging, King Fahad Medical City, Central Second Health Cluster, Ministry of Health, Riyadh, Saudi Arabia; ^2^King Salman Heart Center, King Fahad Medical City, Central Second Health Cluster, Ministry of Health, Riyadh, Saudi Arabia; ^3^Research Center, King Fahad Medical City, Central Second Health Cluster, Ministry of Health, Riyadh, Saudi Arabia

## Abstract

**Background:**

Clinical trials of the COVID-19 vaccine reported the safety and efficacy of mRNA vaccines (AstraZeneca) to help control the disease. Few previous reports have shown various side effects associated with COVID-19 vaccines that vary in severity. The possibility of pericarditis and myocarditis has been observed in people who have received an mRNA COVID-19 vaccine which we are reporting. Acute inflammatory pericarditis can be a rare presentation after receiving the first dose of this vaccine, and it is enriching to share such rare presentations in the era of COVID-19 for better management and outcomes after vaccination. *Case Presentation*. This is a case of acute inflammatory pericarditis with a small pericardial effusion after receiving the first dose of AstraZeneca COVID-19 vaccine in a healthy adult patient who had no other symptoms suggestive of other viral illness in addition to the negative COVID-19 PCR. A 48-year-old healthy male presented nine days after receiving the first dose of COVID-19 AstraZeneca vaccine. The symptoms started three days after the vaccine, when he complained of progressive retrosternal chest pain with low-grade fever and generalized fatigue, followed by exertional dyspnea after a few days. The diagnosis of acute inflammatory pericarditis with small pericardial effusion was established, and the patient was accordingly treated. One week later, the patient showed significant clinical improvement with the resolution of his pericardial effusion. After 39 days, there was a significant radiological resolution of signs of acute pericarditis.

**Conclusion:**

The ongoing COVID-19 outbreak is still under investigation, and guidelines are regularly modified since we are continuously learning about this novel disease, although multiple vaccines have been shown to be effective against COVID-19. However, we report a case of unique clinical manifestation that has not been reported widely in the literature, after receiving the first dose of AstraZeneca COVID-19 vaccine, and that it may help raise awareness of the possible diagnosis and the possibility of inflammatory pericarditis after COVID-19 vaccination.

## 1. Introduction

The viral respiratory infection associated with the novel coronavirus disease (COVID-19) that firstly emerged in Wuhan in China in December 2019 has spread out rapidly among people worldwide [1]. This disease caused a global health crisis, and it has been confirmed as a pandemic and as a Public Health Emergency of International Concern (PHEIC) by the World Health Organization (WHO) [2]. A vaccination campaign against the SARS-CoV-2 virus is the only efficacious intervention to control the outbreak nationally and worldwide [3, 4]. The AstraZeneca vaccine is made up of the adenovirus- (Ad-) vectored AZD1222 (Covishield or Vaxzevria, ChAdOx1-S, Oxford-AstraZeneca) that has been reported to carry active immunization to prevent COVID-19 caused by SARS-CoV-2, in individuals ≥ 18 years old [5]. It has reported a rare side effect such as myocarditis/pericarditis after mRNA COVID-19 vaccines in adults, in several countries including the USA, Israel, UK, Canada, and Italy [6–8]. In this case, we are documenting a rare complication of Oxford-AstraZeneca vaccine which is acute pericarditis that occurs due to inflammation of the pericardium. Pericarditis can be attributed to several factors, including viral, bacterial, or fungal, or it can happen as an isolated condition or as part of a systemic disease [9]. Other possible causes of pericarditis include heart attack, cardiac surgical intervention, and other medical conditions related to injuries and medications. The complications of pericarditis include a spectrum of conditions that varies from mild symptoms like tachycardia to severe symptoms such as cardiac tamponade from the accumulated pericardial effusion [10].

## 2. Case Presentation

A 48-year-old male with an unremarkable medical history presented with retrosternal chest pain aggravated by lying supine and relieved in a sitting position. Also, he had a low-grade fever, fatigue, and exertional dyspnea. The low-grade fever was mildly improving when the patient took paracetamol. The chest pain started three days after he had received the first dose of the COVID-19 vaccine AstraZeneca. A written consent form was obtained from the patient before collecting any information about his medical condition.

**Figure 1:**
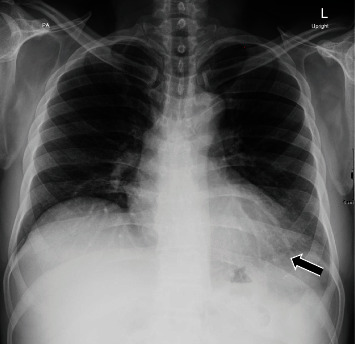
Upright PA chest X-ray showing faint opacity in the lower zone of the left lung.

**Figure 2:**
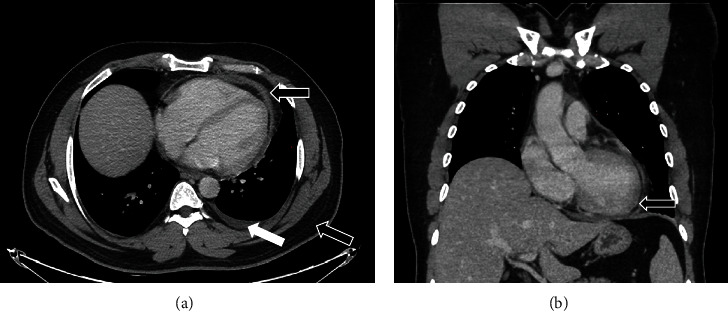
(a) The axial image and (b) the coronal image of the CT scan of the chest post administration of IV contrast showing diffuse pericardial thickening and pericardial fat stranding (black arrow) with small left pleural effusion (white arrow).

**Figure 3:**
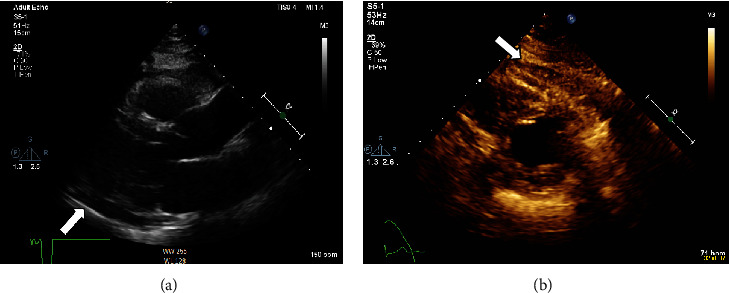
Transthoracic echocardiogram in the long parasternal axis view (a) and short axis view (b).

**Figure 4:**
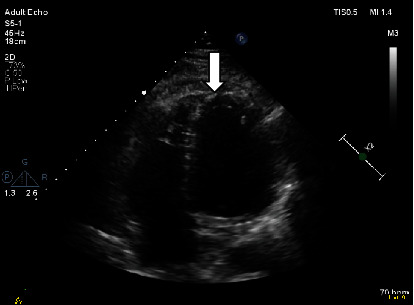
Apical 4 chamber views show pericardial thickening around the apex of left and right ventricles. White arrows; in addition to small pericardial effusion.

**Figure 5:**
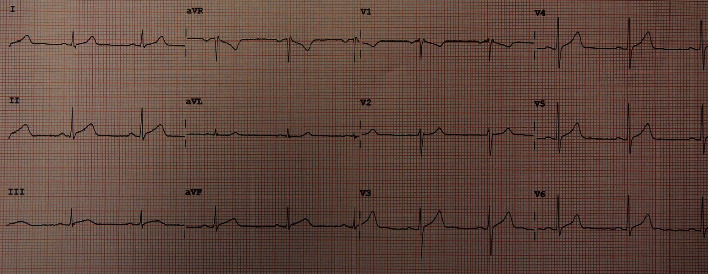
The patient's electrocardiogram showed sinus rhythm, with PR segment elevation in lead aVR (12 lead).

Vital signs and physical examination were within normal limits. Initial laboratory workup including complete blood count, cardiac enzymes, liver function test, and renal function test was within normal limits including a negative COVID-19 PCR. However, the C-reactive protein and the erythrocyte sedimentation rate were elevated reaching 75 mg/L and 77 mm/h, respectively. In addition, the D-dimer test showed a high result reaching 1.44 mg/L. The patient's chest X-ray (Figure 1) showed faint left lower lung zone opacity. Further examination was done on the patient (CT pulmonary angiogram) to rule out pulmonary embolism. CT scan (Figure 2) shows pericardial thickening with surrounding fat stranding and small pericardial and left pleural effusions. After that, an echocardiogram was done (Figures 3 and 4) which showed a small pericardial effusion and pericardial thickening around the apex of the left and right ventricles. No echocardiographic signs of constriction were noted and no echocardiographic evidence of cardiac tamponade. An electrocardiogram (Figure 5) showed sinus rhythm and PR segment elevation in lead aVR. The patient's diagnosis of pericarditis was established due to the patient's symptoms and the relevant data. The initiated treatment plan included ibuprofen of 400 milligrams three times a day for 2 weeks and colchicine of 0.5 milligrams twice a day for 3 months.

## 3. Outcome and Follow-Up

**Figure 6:**
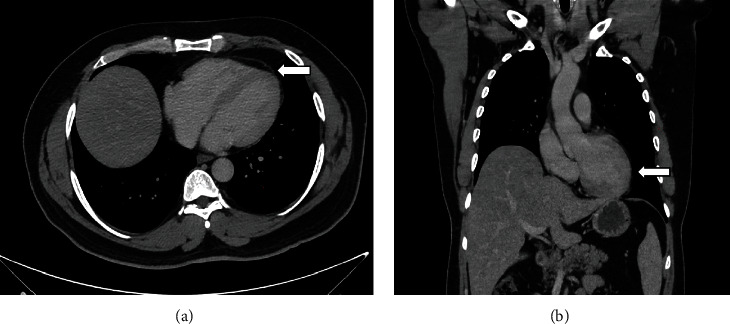
Enhanced CT scan of the chest after a complete course of colchicine and ibuprofen showing improvement of the pericarditis evident by significant improvement of the pericardial thickening and resolution of the pericardial and left pleural effusions.

**Figure 7:**
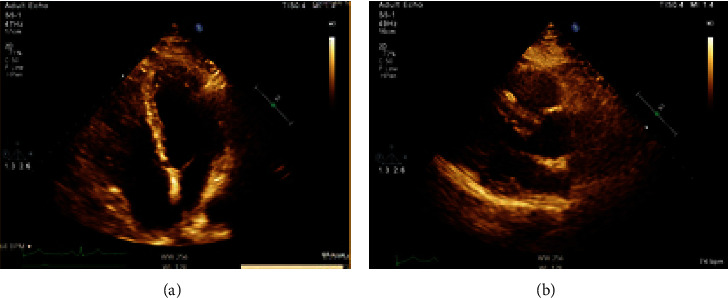
(a) Selected image of transthoracic echocardiogram in apical 4 chamber view and (b) a parasternal long axis view showing marked interval improvement of the pericardial thickening with resolution of the pericardial effusion.

The patient was seen after one week in the cardiology clinic where he showed significant clinical improvement, with the resolution of the chest pain and fever, with minor exertional dyspnea. A timely follow-up with the patient was set after 39 days of the treatment, where he showed total resolution of his symptoms except for mild dyspnea. An enhanced CT scan of the chest (Figure 6) showed marked interval improvement of the pericardial thickening with interval resolution of the pericardial and left pleural effusions. Also, an echocardiogram was done (Figure 7) showing interval improvement of the pericardial thickening with interval resolution of the pericardial effusion, and the C-reactive protein turned to the normal limit.

## 4. Discussion

About 58 vaccines against severe acute respiratory syndrome coronavirus 2 (SARS-CoV-2) have been developed, and in clinical trials, one of them is the AstraZeneca vaccine. [11]. Once the vaccine was approved and put into use, vaccine safety was continuously monitored to identify any serious adverse events. The early safety monitoring of the AstraZeneca vaccine detected multiple postvaccination complications that include anaphylaxis, allergic reactions, thrombotic and embolic events, and immunothrombocytopenia [12, 13]. COVID-19 vaccine AstraZeneca has been studied and approved for its safety and efficacy in preventing severe disease and hospitalization IN 100% as well as preventing symptomatic COVID-19 in 76% and reaching 85% in people aged 65 years and older. The most common side effects of the vaccine are mild to moderate severity while the most serious ones are very rare making the benefits of the vaccine outweigh its risks [12].

The occurrence of pericarditis after immunization is extremely rare, and the number of cases of pericarditis reported after immunization to other vaccines such as influenza and hepatitis B virus is low [14]. On the other, hand when a patient presents with symptoms of acute chest pain to the hospital after receiving the AstraZeneca COVID-19 vaccine, this raises concern for the possibility of pericarditis. This can be further confirmed clinically by typical symptoms of acute pericarditis and reflected by elevation of C-reactive protein, erythrocyte sedimentation rate, D-Dimer, and ECG changes and may be complicated by pericardial effusion. A recent review shows COVID-19 vaccine-related myocardial injury usually presents in young Caucasian males with a median age of 36 years, at 3–10 days postinjection [15]. In our case, symptoms occurred 3 days postvaccination, and the 48-year-old male who complained of continuous chest pain did not need to be hospitalized compared to a similar case in Taiwan [16].

The most common cause of pericarditis is a viral infection which could be direct or secondary immune-mediated. It has been suggested that vaccination may trigger an autoimmune response due to antigenic mimicry as well as due activation of quiescent autoreactive T and B cells [17]. Our case shows the importance of having an index with suspicion of pericarditis during the clinical assessment in any patient who presents with continuous chest pain, fever, and dyspnea a few days postvaccination. COVID-19 itself is associated with a substantially higher risk of myocarditis and other cardiac complications compared with vaccination, and primary infection should be ruled out as the first step before claiming the vaccine as a cause of the acute inflammatory condition. The negative PCR result of the COVID-19 virus and a dramatic improvement in ibuprofen and colchicine were enough to totally exclude COVID-19 infection and consider this acute pericarditis as a side effect of the vaccine, not a complication from COVID-19 infection.

It is difficult to confirm a definitive link between pericarditis and the COVID-19 vaccine; however, a link may exist which is supported by the fact that the patient is a previously healthy male without a previous history of the same symptoms. Additionally, several case reports suggest that COVID-19 infection, COVID-19 vaccine, and other vaccines may trigger an autoimmune reaction [18]. The diagnosis can be established by history and physical examination in addition to the imaging features of pericarditis and pericardial effusion seen in the echocardiography and computed tomography. The appropriate management of this condition can be achieved by the standard treatment of noninfectious pericarditis utilizing Colchicine and nonsteroidal anti-inflammatory drugs. The symptoms are expected to be resolved within two weeks.

## 5. Conclusions

Although pericarditis is emerging as a potential rare adverse effect after COVID-19 vaccination (AstraZeneca), it is rare and the majority of cases of myocarditis/pericarditis reported after mRNA COVID-19 vaccines have occurred within 1-5 days (median 2 days) following the second dose. However, every effort should be made to diagnose early any crucial cardiac complication followed by the COVID-19 vaccination. Although the overall benefit-risk of the COVID-19 AstraZeneca vaccine remains positive, yet the healthcare provider needs to maintain a high index of cardiac symptoms to prevent further consequences.
